# Visuomotor deficiency in *panx1a* knockout zebrafish is linked to dopaminergic signaling

**DOI:** 10.1038/s41598-020-66378-y

**Published:** 2020-06-12

**Authors:** Nickie Safarian, Paige Whyte-Fagundes, Christiane Zoidl, Jörg Grigull, Georg Zoidl

**Affiliations:** 1 Department of Biology, York University, Toronto Ontario, M3J1P3 Canada; 2 Department of Mathematics and Statistics, York University, Toronto Ontario, M3J1P3 Canada; 3 Center of Vision Research, York University, Toronto Ontario, M3J1P3 Canada

**Keywords:** Molecular biology, Neuroscience

## Abstract

Pannexin 1 (Panx1) forms ATP-permeable membrane channels that play roles in the nervous system. The analysis of roles in both standard and pathological conditions benefits from a model organism with rapid development and early onset of behaviors. Such a model was developed by ablating the zebrafish *panx1a* gene using TALEN technology. Here, RNA-seq analysis of 6 days post fertilization larvae were confirmed by Real-Time PCR and paired with testing visual-motor behavior and *in vivo* electrophysiology. Results demonstrated that loss of *panx1a* specifically affected the expression of gene classes representing the development of the visual system and visual processing. Abnormal swimming behavior in the dark and the expression regulation of pre-and postsynaptic biomarkers suggested changes in dopaminergic signaling. Indeed, altered visuomotor behavior in the absence of functional *Panx1a* was evoked through D1/D2-like receptor agonist treatment and rescued with the D2-like receptor antagonist Haloperidol. Local field potentials recorded from superficial areas of the optic tectum receiving input from the retina confirmed abnormal responses to visual stimuli, which resembled treatments with a dopamine receptor agonist or pharmacological blocking of *Panx1a*. We conclude that *Panx1a* functions are relevant at a time point when neuronal networks supporting visual-motor functions undergo modifications preparing for complex behaviors of freely swimming fish.

## Introduction

Pannexin 1 (Panx1) is an integral membrane glycoprotein forming ATP release channels in different tissues and cell types^1–5^, including neurons^6–8^. In the CNS, evidence for physiological functions of Panx1 points at roles in the processing of sensory signals and learning and memory^9–11^. For example, in Panx1 knockout mice, altered retinal contrast sensitivity^12^ and hearing loss have been found^13,14^. Also, performance in spatial learning and memory abilities such as object recognition and fear conditioning tasks are decreased^9,15,16^. Intellectual disabilities, severe hearing loss, primary ovarian failure, kyphoscoliosis, and difficulties navigating in darkness were found in the first human patient identified with a homozygous Panx1 mutation^17^.

To form a better view of Panx1 functions in the processing of sensory information, we used the zebrafish as model organisms. Two Panx1 genes, *panx1a* and *panx1b*, originated from partial genome duplications during early teleost evolution^18,19^. Although the two genes have been separated for more than 200 million years, principal channel functions are comparable to rodent or human Panx1^20^. In the retina, the *panx1a* protein is expressed in horizontal cells^19,20^ and plays essential roles in feedback from horizontal cells to cones in adult zebrafish^21–23^. Here the *panx1a* gene was edited using transcription activator-like effector nucleases (TALEN). A loss of function mutation allowed to investigate *Panx1a* in 6 dpf old zebrafish larvae at a developmental stage when neuronal networks for visually guided locomotor behaviors were functional. Transcriptome analysis detected noteworthy expression differences of genes associated with eye development and vision-related processes. When followed up, altered dopaminergic signaling affecting both pre- and postsynaptic proteins were found. The molecular evidence was supported by measuring abnormal responsiveness of *panx1a*^−/−^ larvae to darkness, or after the abrupt loss of illumination. Pharmacological activation of D1/D2-like dopamine receptors simulated this phenotype. Blocking D2-like receptors with Haloperidol rescued the phenotype. *In vivo* electrophysiological recording of local field potentials (LOF) from the larval optic tectum in a region receiving input from the retina demonstrated that the dynamic transition from low to higher-frequency brain waves in light and darkness was compromised in *panx1a*^−/−^ larvae. This phenotype was reproduced by pharmacological blocking of *Panx1a*, or by treatment with the D1/D2 receptor agonist apomorphine. This research delivers a novel association between *Panx1a* in the integration of sensory-motor behavior through modulation of dopaminergic signaling.

## Results

### Targeted ablation of *panx1a*

**Figure 1:**
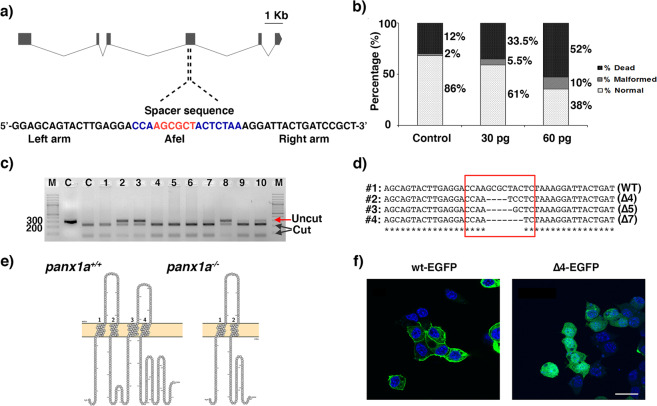
Generating *panx1a*^−/−^ fish using TALENs – (**a**) The zebrafish *panx1a* gene structure with six coding exons. The position of the left and right TALENs sequence with the spacer sequence and AfeI restriction site in blue and red is highlighted. (**b**) Larval survival rates (in %) one day after (1 dpf) microinjection. A dose of 30 pg TALENs pair resulted in more than 50% survival rate and was selected for the experiments generating the *panx1a*^−/−^ fish line. (**c**) The RFLP-assay shows the loss of the AfeI recognition sequence (indicated by uncut) in four out of ten F0 larvae tested. (**d**) A sequencing alignment is demonstrating small deletions causing frameshift mutations found in three different F0 larvae. (**e**) The predicted sequence features of the mutated *panx1a* were visualized using the Protter open-source tool (wlab.ethz.ch/protter). A 4 bp deletion in *Panx1a* exon 4 resulted in a frameshift causing a premature stop codon at amino acid 195. (**f**) Localization of the truncated *Panx1a* protein – Confocal images of transiently transfected proteins *Panx1a*^wt-EGFP^ (left panel) and *Panx1a* ^Δ4-EGFP^ (right panel) in Neuro2a cells. Nuclei were stained with DAPI. Scale bar: 10 μm.

The *panx1a* mutant allele was generated by TALEN-mediated mutagenesis targeting the single *AfeI* restriction endonuclease recognition site in the fourth exon of *panx1a* (Fig. 1a). Three doses of 30, 60, and 100 pg/TALEN cRNA pair were injected into 1-cell stage embryos of the TL strain. 61% and 38.6% of all F0 embryos developed typically after injection of 30 pg/nl and 60 pg/nl cRNA, whereas 90% of the embryos developed malformations or died within 24hrs after injection of 100 pg/nl **(**Fig. 1b**)**. A concentration of 30 pg/nl cRNA was used in follow-up gene-targeting experiments. The restriction fragment length polymorphism test (RLFP RFLP) of ten randomly selected embryos revealed a mutagenesis efficiency of ≈50%, as evidenced by a partial loss of the restriction enzyme recognition site at the TALENs cut sites (Fig. 1c). DNA sequence analysis of multiple microinjected embryos confirmed the efficient introduction of short, 4 to 7 long nucleotide deletions in the *panx1a* exon 4 (Fig. 1d). A founder fish carrying a four bp deletion (*panx1a*^Δ4^) was selected for further experimentation. The four base pair deletion caused a frameshift at amino acid 195, resulting in a premature stop codon leading to truncated 201-amino-acid protein, lacking most of the 416 amino acid long *Panx1a* protein sequence including two transmembrane regions and the entire carboxyterminal domain (Fig. 1e). After transfection into mouse Neuroblastoma 2a (Neuro2a) cells the subcellular localization of the truncated *Panx1a*^Δ4-EGFP^ protein had a diffuse cytoplasmic signal suggesting that the mutant protein was unable to traffic efficiently to the cell membrane (Fig. 1f), and unlikely to form functional channels (Fig. 1f, left panel). The full-length *Panx1a*^wt-EGFP^ protein was detectable in the plasma membrane (Fig. 1f, right panel) in line with previous reports^20,24^.

### Characterization of *panx1a*^−/−^ larvae

**Figure 2:**
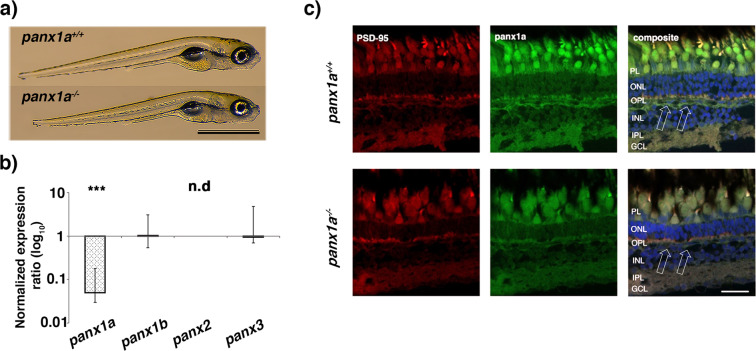
Phenotypic characterization of *panx1a*^−/−^ larvae – (**a**) Age-matched wild type TL and ^panx1a^^−/−^ larvae (6 dpf) showing regular morphology. (**b**) RT-qPCR analysis of pannexin expression in 6 dpf larva. (**c**) The *panx1a* expression is reduced in the adult *panx1a*^−/−^ zebrafish. The expression was determined using an affinity purified rabbit anti-*panx1a* antibody directed against the carboxy-terminal 129 amino acids of the *Panx1a* protein. Detection of the PSD-95 protein in cone terminals served as an internal control. Cell nuclei were stained with DAPI. In wild type TL fish arrows indicate *Panx1a* immunoreactivity found in the horizontal cell layer as reported previously^19,20^. The immunoreactivity was significantly reduced in *panx1a*^−/−^ fish. Residual staining was overlapping with DAPI stained nuclei and considered as unspecific. Abbreviations: PL, photoreceptor layer; ONL, outer nuclear layer; OPL, outer plexiform layer; INL, inner nuclear layer; IPL, inner plexiform layer; GCL, ganglion cell layer. Scale bar 100 µm. n.d., Not detected. Significance: ***p-value < 0.001.

A comparison of *panx1a*^*−/−*^ and wild type TL larvae (F3 generation) revealed no gross anatomical defects (Fig. 2a), and juvenile fish were visually indistinguishable from age-matched TL siblings. Adult *panx1a*^−/−^ zebrafish were viable and fertile like the parental TL strain, or heterozygous fish, suggesting that developmental differences were restricted to early developmental stages. The *panx1a* mRNA level was significantly reduced in 6 dpf *panx1a*^−/−^ larvae indicating small deletion-mediated RNA decay **(**Fig. 2b**)**. Compensatory regulation of three other pannexin genes similar to previous reports using Panx1^−/−^ mouse models was excluded^25^. *Panx1b* mRNA was expressed at equal levels in both wild-type and *panx1a*^−/−^. A low level of *panx3* mRNA expression was detected, but not *panx2* mRNA.

Immunohistochemistry (IHC) experiments confirmed a reduction of *Panx1a* expression in the retina of adult zebrafish. PSD-95 staining of cone photoreceptor terminals was similar in both genotypes. No gross anatomical alteration of the retina was noticeable. The immunoreactivity found in the outer retina of *panx1a*^+/+^ controls was reduced in age-matched knock-out retina (Fig. 2c). *Panx1a* expression in the outer retina of 3 dpf larvae TL larvae resembled the localization found in the adult retina (see Supplementary Fig. S1a). The *Panx1a* immunoreactivity was reduced in *Panx1a*^−/−^ larvae (Supplementary Fig. S1b). Residual immunoreactivity found in both adult and 3 dpf retina was attributed to cross-reactivity of the antibody with the *panx1b* protein, which shares 66% amino acid identity with *panx1a* in the carboxyterminal domain.

### Transcriptome profiling of 6 dpf *panx1a*^−/−^ larvae

**Figure 3:**
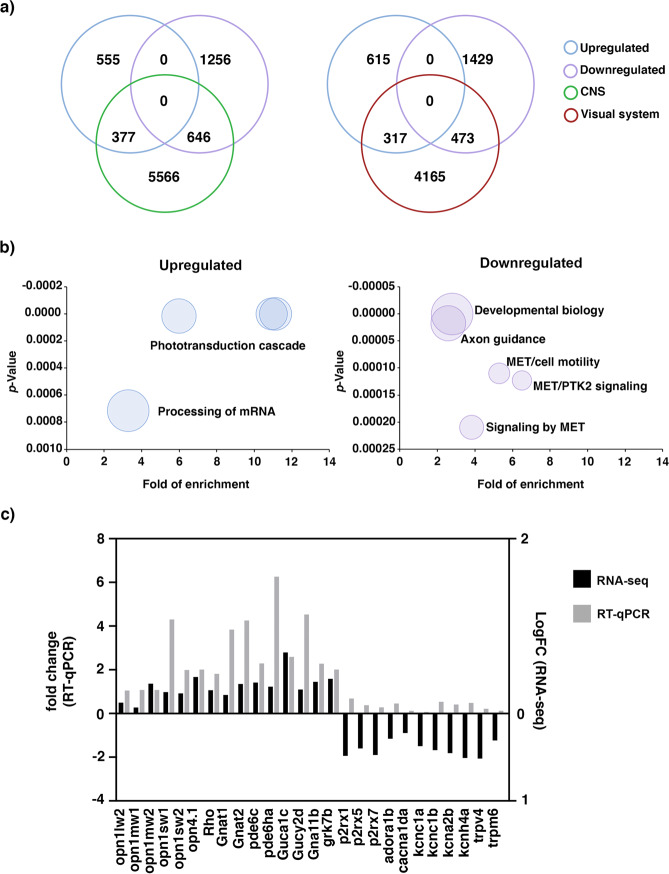
RNA-seq analysis of 6 dpf *panx1a*^−/−^ larvae – (**a**) Comparison of deregulated genes against two categories: central nervous system (CNS) and the visual system. (**b**) GO annotation of RNA-seq data: the upregulated genes were enriched in the phototransduction cascade and mRNA processing pathways. The downregulated genes were annotated for developmental processes and signaling pathways. (**c**) Validation of RNA-seq data by RT-qPCR.

The transcriptomes of *panx1a*^−/−^ and wild-type TL larvae were compared at 6 dpf (NCBI Gene Expression Omnibus (GEO) database; data deposit GSE147068;). A total of 12302 RNAs was found in each of the six samples (TL control, n = 3; *panx1a*^−/−^, n = 3) sequenced. In *panx1a*^−/−^ larva, 1902 RNAs were downregulated, and 933 RNAs upregulated when the cutoff for the false discovery rate (FDR) was set to 0.05, and the significance of regulation was defined as *p-*value < 0.0001. Gene-specific expression information retrieved from the Zebrafish Information Network (ZFIN) database allowed categorizing the representation of regulated genes to those previously described as expressed in the central nervous system (CNS) or visual system. Three hundred seventy-seven upregulated genes and 646 downregulated genes matched genes expressed in the central nervous system (Fig. 3a, left panel). Furthermore, 317 upregulated genes and 473 downregulated genes matched genes expressed in the visual system (Fig. 3a, right panel).

The AmiGO2 analysis (http://amigo.geneontology.org/amigo/landing) revealed enriched Gene Ontology (GO) terms and pathways (Supplementary Tables S1a and S1b). The most significant enriched Reactome processes (cut-off: *p-*value < 0.001; fold change of expression >2.5) (Fig. 3b) represented the phototransduction cascade, as well as the processing of mRNAs (Fig. 3b. left plot, upregulated genes). Processes such as development and cell signaling through membrane receptors represented downregulated genes (Fig. 3b, right plot).

RNAseq data (cutoff: *p-*value < 0.0001; FDR 0.05) were also validated by RNA-qPCR (cutoff: *p*-value < 0.05). This experiment confirmed that targeting *panx1a* upregulated mRNAs specific for phototransduction and the development of the visual system (Fig. 3c, Supplementary Table S2). Selected purinergic receptors and genes representing voltage-gated ion channel families were confirmed as downregulated in *panx1a*^−/−^ larva **(**Fig. 3c, Supplementary Table S2**)**. We concluded that the genetic ablation of *panx1a* in the zebrafish caused a molecular phenotype in which both the visual system and central nervous system was affected in 6 dpf old larvae.

### Loss of *panx1a* impairs locomotion in the dark

**Figure 4:**
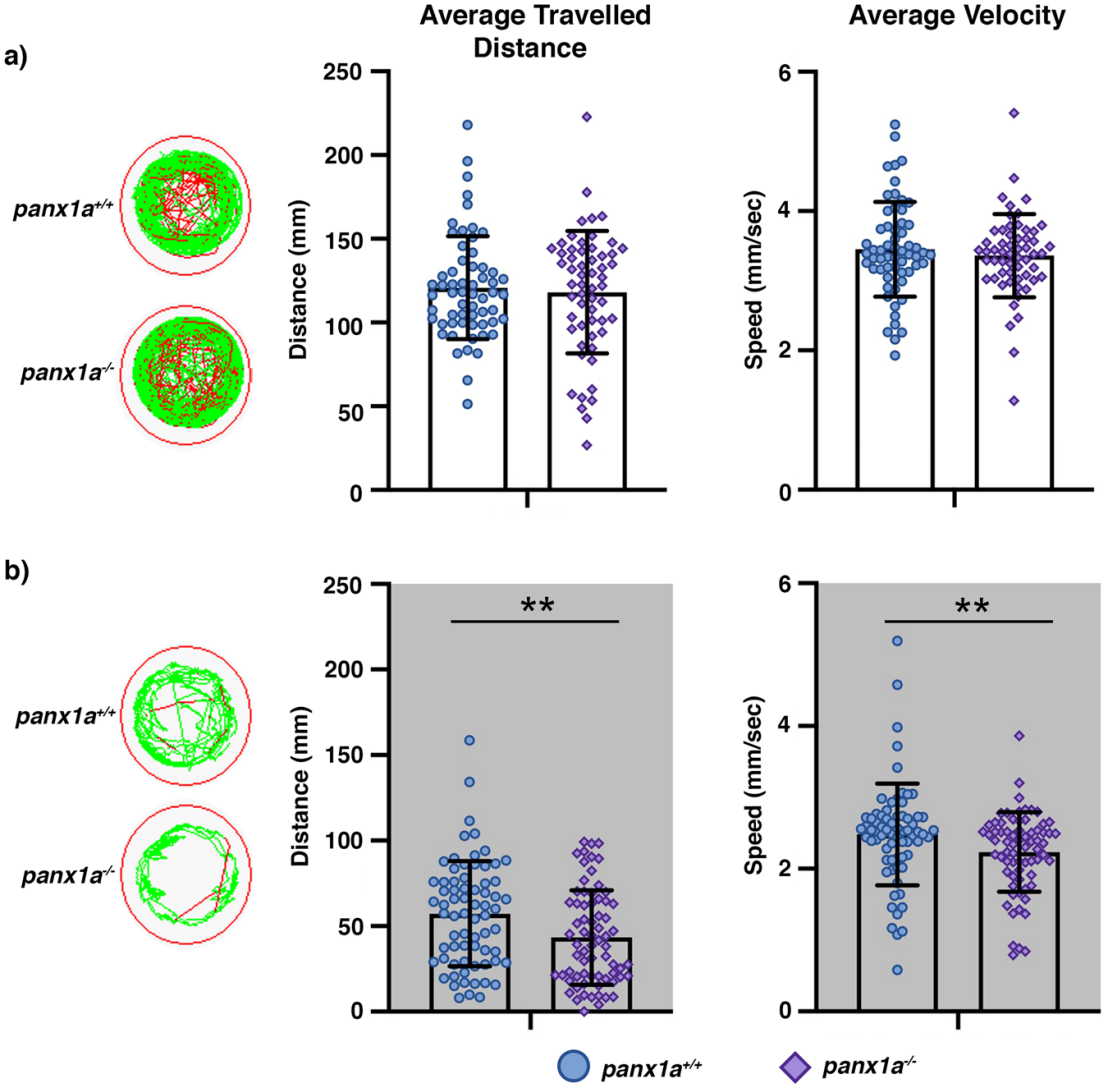
Locomotor activity in Light-Dark conditions – Locomotion was video tracked for 60 min in (**a**) Light-ON and (**b**) Light-OFF conditions. The wells on the left show examples of *panx1a*^−/−^ and *panx1a*^+/+^ locomotion patterns. Medium (<20 mm/sec) or high-speed movements (>20 mm/sec) are visualized with green and red colors. Graphs demonstrate the averaged traveled distance (in mm) and the average velocity (mm/sec) of n = 60 larvae for each genotype. Significance: *p-value < 0.01.

Visually guided behavior was investigated after transcriptome analysis suggested changes to visual functions. At 6 dpf, both genotypes, *panx1a*^+/+^ and *panx1a*^−/−^, swam longer distances and more rapidly during the Light-ON phase than during the Light-OFF phase when baseline swimming activity of unrestrained larvae was tested. In light, both genotypes showed no significant difference in the average distance traveled (t = 1.86, df = 133.52, *p*-value = 0.06531; n = 60) and average velocity (t = 1.03, df = 133.74, *p*-value = 0.3065; n = 60) (Fig. 4a**)**. A representative example illustrates the preference of larvae for swimming close to the circumferences of the wells, with spontaneous crossings of the central zone. During the dark phase, *panx1a*^−/−^ larvae were less active and showed intermittent episodes of swimming bouts with slow and medium speeds. *Panx1a*^−/−^ larvae swam significantly less (t = −3.08, df = 135.9, *p-*value = 0.002; n = 70) and with lower velocity (t = −2.79, df = 131.73, *p*-value = 0.006; n = 70) compared to TL-controls (Fig. 4b). Furthermore, *panx1a*^−/−^ larvae avoided crossing the central zone of the well in the dark.

### Visual-motor response (VMR) in *panx1a*^−/−^ larvae

**Figure 5:**
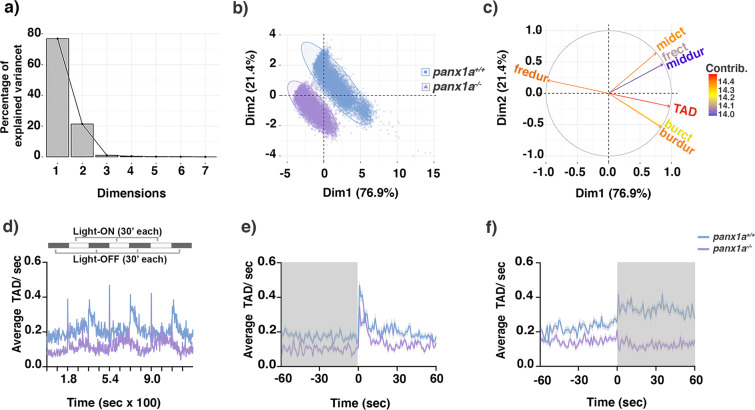
Visual-motor response (VMR) changes in *panx1a*^−/−^ larvae – (**a**) Principal component analysis (PCA) transforming the VMR multidimensional data. The scree plot shows that Dimensions 1 and 2 capture more than 76% and 21% of the data variance. (**b**) Mapping individual samples on a two-dimensional space revealed that the two genotype groups were separated into two distinct clusters. (**c**) The variable correlation plot represented the coordinates of the seven variables (i.e., freeze counts(frect) and duration (fredur), medium activity counts (midct) and duration (middur), hyperactivity counts (burct) and duration (burdur), and the total activity duration (TAD)) in the first two dimensions. The variables were colored from blue to red as their contribution to PCs increases. Total activity duration (TAD; in color red) showed the highest contribution to the variability in Dimension 1 and was chosen to visualize differences between genotypes. (**d**) The outline of the experimental paradigm is shown on top. The line graph shows the average TAD of *panx1a*^−/−^ and *panx1a*^+/+^ larvae from a representative test with n = 24 larvae for each genotype. The activity was defined as the fraction of frames per second that a larva spent swimming (see methods for the detailed information). (**e,f**) The results for average Light-ON and Light-OFF VMR are shown from 1 min before the light switch to 1 min after the light switch. The color ribbons surrounding the average activity line graph correspond to 1 S.E.M. The values are the average of the second trial from four independent tests with n = 96 larvae for each genotype. The Light-ON and Light-OFF periods are indicated by white and black bars at the top of the panels.

The visual-motor response (VMR) assay measured the responsiveness of zebrafish larvae to light changes. The orthogonal transformation of the multidimensional VMR data by principal component analysis (PCA) was used to identify the most relevant of seven behavioral parameters. PC1 and PC2 captured more than 76% and 21% of the data variance (Fig. 5a). Two-dimensional PCA plotting confirmed that both genotypes were separated into two distinct clusters (Fig. 5b). The variable correlation plot allowed to conclude that the total activity duration (TAD; in red) had the most significant contribution to the variability in PC1 (Fig. 5c). Thus, TAD was chosen to determine the differences between genotypes in VMR assays.

TL control and *panx1a*^−/−^ larvae showed distinct VMR, as demonstrated by the corresponding average TAD plots in an experimental paradigm of 3 cycles of alternating Light-ON and Light-OFF periods (Fig. 5d**; top panel**). In all cycles, the larval activity was consistently reduced in the absence of *Panx1a* (Fig. 5d). In the 1 min period before the Light-ON stimulus (−60-0 s), *panx1a*^−/−^ larvae showed a significantly lower activity level than TL controls (*p*-value = 0.015). At Light-ON (0–1 sec), both TL controls and mutant larvae augmented their activity immediately; only the peak response to the light stimulus was trending lower in *panx1a*^−/−^ larvae compared to TL controls (*p*-value = 0.382). During the subsequent light period, both mutants and TL controls gradually returned to the baseline activity level (in 1–60 sec, *p*-value = 0.876; Fig. 5e).

Notably, at Light-OFF, the mutants responded differently to control TL larvae. For the 1 min period before Light-OFF stimulus (−60-0 s), *panx1a*^−/−^ larvae had lower baseline activity (*p*-value = 0.036). Following the abrupt loss of illumination (0–1 s), TL control larvae initially increased their locomotor activity and then remain more active for 10 ± 2 minutes. After that, the magnitude and duration of swim bouts gradually returned to baseline activity level. In contrast, *panx1a*^−/−^ larvae demonstrated a significant reduction (*p*-value = 0.001) of dark stimulus-response and continued to show a reduced activity during the dark period. This observation was supported by the corresponding TAD plots (Fig. 5f), in which the activity of mutants after the light change was noticeably lower when compared to TL controls (*p*-value = 0.0249). These results indicated that the *panx1a* mutant fish readily detected the Light-ON stimulus but responded weakly to the Light-OFF condition. Therefore, *Panx1a* functions appear important for the detection of light decrement and the corresponding behavioral response in larval zebrafish.

### Alterations of the Dopamine (DA) signaling pathway in *panx1a* mutants

**Figure 6:**
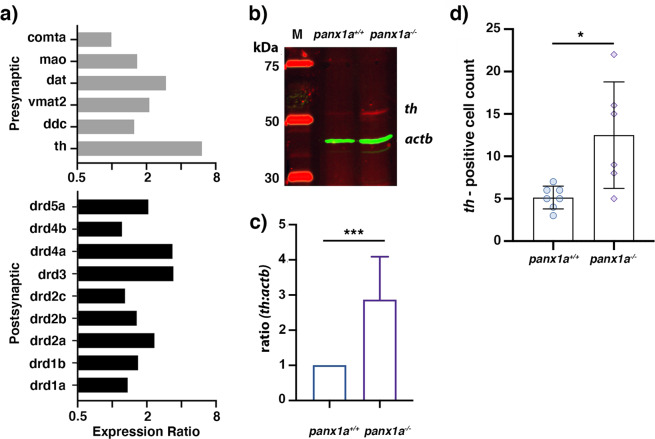
Expression modulation in the dopaminergic pathway – (**a**) RT-qPCR quantification of expression changes. Genes are arranged by typical localization in a dopaminergic synapse. (**b**) Western blot analysis of protein lysates from whole 6 dpf larvae showing tyrosine hydroxylase (th) immunoreactivity and the beta-actin control (actb). On the bottom right, quantification of th:actb ratio for n = 4 independent experiments. (**c**) Quantification of th-positive cells in the retina of wild-type (n = 7) and mutant (n = 6) larvae (3 dpf). Mean ± SD. Significance: *p -value < 0.05, ***p -value < 0.001.

The role of dopaminergic signaling was investigated based on the differential expression of dopamine receptors found by RNA-seq and altered locomotion in the dark. These observations suggested that loss of *Panx1a* function in knock-out larvae could have affected expression of genes involved in dopamine signaling. This idea was tested first by RT-qPCR. Among transcripts of dopaminergic genes located in the presynaptic compartment, a significant up-regulation of the tyrosine hydroxylase gene, which catalyzes the rate-limiting step in DA synthesis, was detected when cut-off criteria for the expression ratio was >1.5, and the *p*-value < 0.05. Other prominent regulated transcripts were *slc6a3/dat*, a sodium-dependent dopamine transporter, and *slc18a2/vmat2*, an ATP-dependent vesicular monoamine transporter involved in the dopamine neurotransmitter release cycle **(**Fig. 6a**;** Supplementary Table S3**)**. Dopamine receptor genes significantly upregulated in *panx1*^−/−^ larvae represented both D1/D2 subfamilies (Fig. 6a; Supplementary Table S3). A three-fold upregulation of the tyrosine hydroxylase protein in *panx1*^−/−^ larvae (Fig. 6b,c; n = 4; significance *p*-value < 0.01), and a significantly higher *Th*-positive cell count in the retina of *panx1a*^−/−^ larvae (Fig. 6d; *panx1a*^+/+^, n = 7; panx1a^−/−^, n = 6; *p*-value = 0.034; Supplementary Fig. S1c–e) complemented the RT-qPCR result.

Apomorphine (Apo), a non-specific dopamine receptors agonist, was used to investigate the overall role of augmented dopamine signaling in *panx1a*^−/−^ and control larvae. Dose-response assays confirmed the known U-shape relationship between Apo concentrations and larval activity in the light and dark (Supplementary Fig. S2a and S2b)^26,27^. A concentration of 50 µM Apo, representing the most substantial effect, was selected. The larval activity during the second trial was analyzed (i.e., 90–120 min), when the maximal effect of Apo was reached.

**Figure 7:**
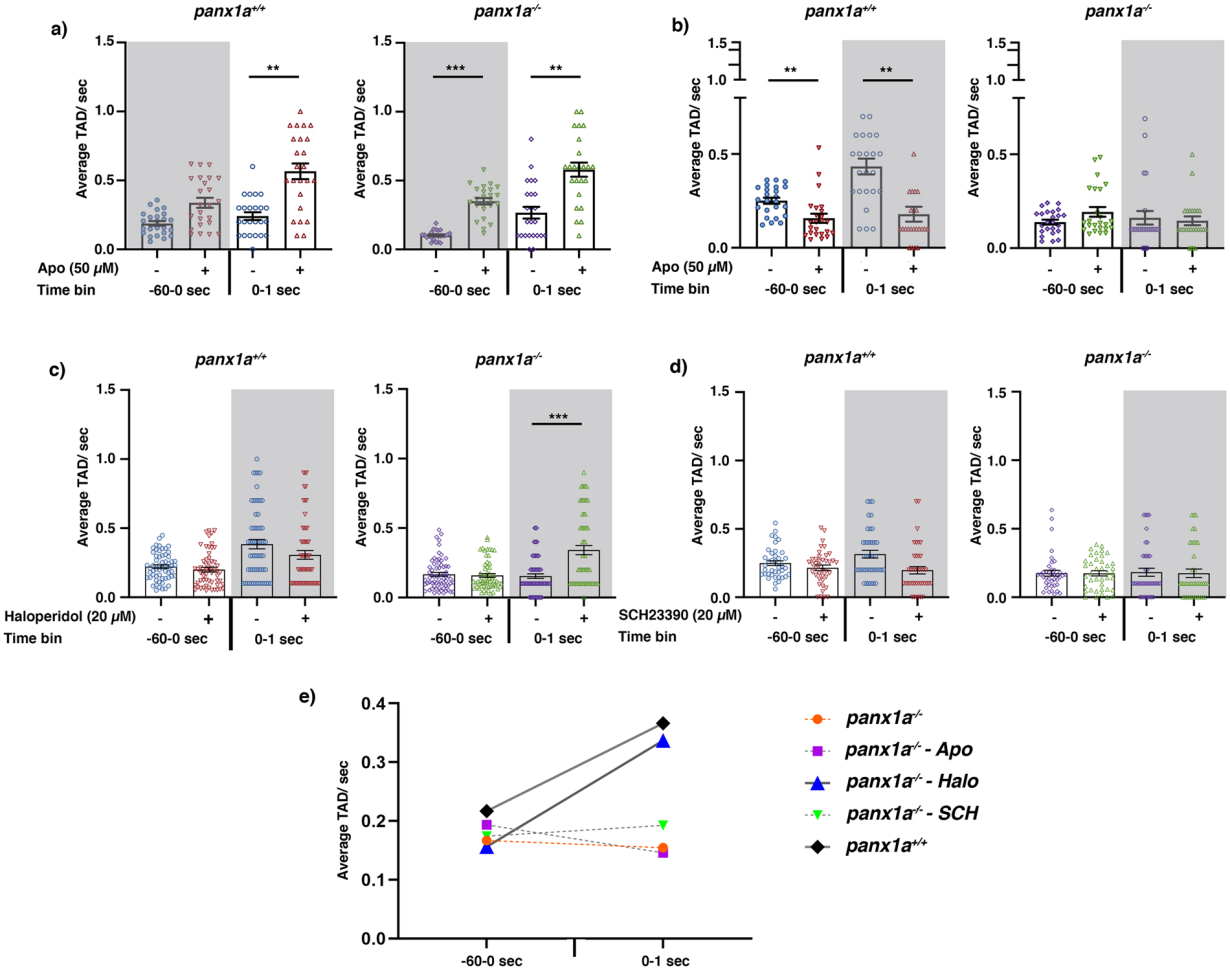
Dopaminergic signaling modulates larval visuomotor activity – The average (**a**) Light-ON and b-d) Light-OFF VMR from 1 min before the light switch to 1 sec after the light switch is graphed as mean ± SEM for *panx1a*^+/+^ controls (left panels) and *panx1a*^−/−^ (right panels). (**a**) Apomorphine (a D1/D2-receptor agonist; 50 μM) significantly increases the Light-ON peak response in both genotypes (n = 68). (**b**) The suppression of the Light-OFF response in wild-type larvae was lost in mutants after Apo treatment (n = 68). (**c**) Haloperidol, a D2-like receptor antagonist (20 μM) significantly increases the Light-OFF peak response in mutants (n = 64); however, it does not affect the peak response in the wild-type larvae (n = 60). (**d**) Treatment with SCH-23390, a D1-like receptor antagonist (20 μM) slightly attenuated the Light-OFF response in wild-types (n = 40), with no significant changes on the behavioral phenotype of *panx1a*^−/−^ animals (n = 40). e) Changes in the average TAD at the Light-OFF were compared between mutants under different treatments and wild-type larvae with no treatment. The graph illustrates that Haloperidol effectively rescued the Light-OFF phenotype of *panx1a*^−/−^larvae. Significance: *p -value < 0.05, ***p -value ≤ 0.001.

Figure 7a exemplifies larvae of both genotypes, which appeared to be hyperactive in the dark period preceding the Light-ON (i.e., −60 to 0 sec). Though, the increment of activity was significant only in mutants. Also, Apo treatment affected the Light-ON peak response (0 to 1 sec period) similarly in both groups. It significantly raised the magnitude of the Light-ON peak response, so no further difference was detected between groups (All the *p*-values are given in Table 1).

**Table 1:** The multivariate comparisons of VMR between Apo-treated and untreated larvae.

Typ of stimulus	Type of treatment	Control	Treatment (Apomorphine (50 µM))		
	Genotype Time bin	+/+: −/−	+/+	−/−	+/+: −/−
Light-ON	Before (−60-0 S)	3.43e^−07^	0.552	5.17e^−08^	0.901
	At (0–1 S)	0,615	0.078	3.14e^−06^	0.481
Light-OFF	Before (−60-0 S)	4.91e^−08^	2.18e^−03^	5.74e^−04^	9.16e^−03^
	At (0–1 S)	3.408e^−03^	4.86e^−03^	0.787	0.974

When the larval activity was analyzed during the second Light-OFF trail, differing patterns of effects were observed in the two groups. In the light period preceding dark (i.e., −60 to 0 sec), Apo (50 µM) markedly decreased activity in TL controls but did not significantly alter that in *panx1a*^−/−^ larvae (Fig. 7b). At the light offset (0 to 1 sec), similar behaviors occurred. Apo abolished the larval peak response to the sudden darkness in TL controls to the point that it resembled a mutant response. No significant difference was observed in the *panx1a*^−/−^ larvae Light-OFF peak response (Fig. 7b**;** All the *p*-values are given in Table 1).

The mean activity data suggested that changes in the lighting conditions affected the levels of activity of larvae given 50 µM Apo (Fig. 7a,b). While Apo treatment enhanced larval reaction to the sudden illumination (Light-ON) regardless of their genotypes, it suppressed larval response to the sudden darkness (Light-OFF) only in *panx1a*^+/+^.

Stimulation of D1/D2 dopaminergic signaling with Apo induced in *panx1a*^+/+^ larvae a *panx1a*^−/−^ like Light-OFF response. This result suggested a causal relationship between *Panx1a* function, augmentation of dopaminergic signaling, and the described behavioral phenotype. The class of dopamine receptor which modulated the Light-OFF component of the VMR was identified using D1- and D2-like dopamine receptor antagonists. Treatment with the D2-like receptor antagonist Haloperidol (20 μM) rescued the dopamine-induced Light-OFF response deficiency in mutants (n = 64; *p*-value < 0.001) (Table 2), with no significant effects in wild-type controls (n = 64; *p*-value = 0.27) (Fig. 7c). The D1-like receptor antagonist SCH-23390 (20 μM) did not affect the mutants’ peak response (n = 40; p-value = 0.76) and the Light-OFF response in wild-type control larvae (n = 40, p-value = 0.218) (Fig. 7d). A comparison of TAD at Light-OFF across different treatments showed that Haloperidol effectively rescued the Light-OFF phenotype of *panx1a*^−/−^ larvae (Fig. 7e). The results suggested a participation of D2-like receptors in dopamine-induced Light-OFF response deficiency observed in *panx1a*^−/−^ zebrafish larvae.

**Table 2:** The multivariate comparisons of the VMR (Light-OFF) for Haloperidol- and SCH-23390-treated larvae.

Type of treatment	Genotype Time bin	Control	Treated		
Antagonists		+/+: −/−	+/+	−/−	+/+: −/−
Haloperidol (20 µM)	Before (−60-0 S)	0.017	0.428	0.942	0.318
	At (0–1 S)	<0.0001	0.271	0.0001	0.825
SCH23390 (20 µM)	Before (−60-0 S)	0.018	0.972	0.998	0.857
	At (0–1 S)	0.017	0.218	0.999	0.238

### Loss of *panx1a* modifies brain wave frequency transitions in response to light

**Figure 8:**
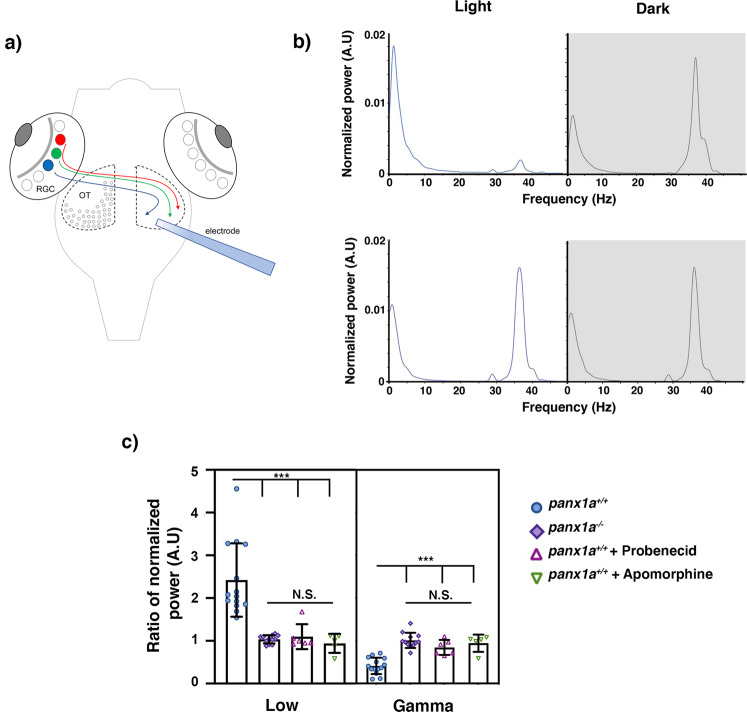
*Panx1a* modulates local field potentials in the optic tectum. (**a**) Outline of the recording setup showing the typical placement of electrodes in the optic tectum (OT) in a region where axons from retinal ganglion cells (RGC) terminate. (**a**) Examples of *in vivo* recordings from the optic tectum of a *panx1a*^+/+^ control during Light-ON. Normalized power for low (<10 Hzhz) and gamma frequencies (30–45 Hz) is detected. In Light-OFF a shift to gamma frequency power is observed (Grey). (**b**) This transition between in Light-ON (Purple) and Light-OFF (Grey) conditions is lost in *panx1a*^−/−^ larvae. (**c**) Quantification of changes in normalized power ratios for low (Left) and gamma (Right) frequencies in response to light conditions. Knocking out *panx1a* (n = 12) and blocking *Panx1a* with Probenecid (n = 6), or Apomorphine treatment targeting D1/D2 receptors (n = 5), results in a significant loss in the regulation of the transition of frequencies compared to *panx1a*^+/+^ (n = 15). Significance: n.s not significant, ***p -value ≤ 0.001.

Changes to retinotectal circuitry were tested by recording local field potentials (LOF) in the optic tectum of immobilized 6 dpf larvae exposed to full-field light stimuli (Fig. 8a). In wild-type larvae, the peak in normalized power shifted from low frequencies (less than 10 Hz) to gamma frequencies (35–40 Hz) when the light was shut off, and larvae were exposed to darkness for 10 minutes (Fig. 8b). This response was abolished in *panx1a*^−/−^ larvae. Shifting from dark to light conditions gave identical results in peak power (Fig. 8b). Loss of *Panx1a*, as well as treatment of wild type larvae with 100 μM probenecid, an established blocker for pannexins, significantly reduced the changes in normalized power for both the low and gamma frequency ranges compared to control TL larvae (Fig. 8c, Table 3). Further, the treatment of wild-type larvae with 50μM apomorphine, the D1R/D2R agonist, created a significant change to transitions between low and gamma frequencies under light stimulus when compared to controls (Fig. 8c, Table 3). Our results demonstrated that targeting *Panx1a*, either with pharmacology or by gene-editing, or targeting dopaminergic synapses, caused a significant change in properties of the retinotectal pathway.

**Table 3:** The t-Test of power spectral density between groups.

Frequency	Genotypes	Control	Apomorphine (50 μM)	Probenecid (100 μM)
Low	+/+: −/−	1.068e-05	−	−
	+/+	−	0.0016	0.0019
	−/−	−	0.225	0.511
Gamma	+/+: −/−	2.012e-08	−	−
	+/+	−	6.14e-05	1.65e-04
	−/−	−	0.524	0.082

## Discussion

Ablation of the *panx1a* gene caused profound differences of the transcriptome, visual-motor behavior, as well as vision-related neuronal network properties in the optic tectum of 6 dpf zebrafish larvae. The employed targeted gene knockout approach, transcription activator-like effector nucleases (TALEN), introduced a small deletion in exon4 of the *panx1a* gene. This mutation caused the generation of a truncated protein. The mutation was distinct from the previously reported *panx1a*^−/−^ model in which the start codon was targeted^23^, but better compared to the mouse knockout model with deletions of exons3 and 4, which was used previously to investigate sensory processes and learning and memory^9,28^. Similar to other rodents^29,30^ and the other zebrafish model^23^, the *panx1a*^−/−^ zebrafish are viable and fertile, with no apparent anatomical abnormalities or morphologic changes to the eyes.

All investigations were performed at 6 dpf when the subdivisions of the brain and sensory systems have formed, and excitatory glutamatergic neurons, inhibitory GABAergic interneurons, astrocytes, and microglia required for normal excitatory discharges within neuronal networks are functional^31–33^. The transcriptome analysis demonstrated that loss of *panx1a* effectively altered transcription of almost 20% of all expressed genes. The observed expression regulation was consistent with the known expression profile of *panx1a* across multiple organ systems^18–20^. Vision-related processes and the development of the eye, or the transportome, a collective term referring to proteins facilitating transport to and across membranes, which includes ion channels, purinergic and neurotransmitter receptors, solute carrier (SLC) transporters were affected^34^. These transcriptional changes are noteworthy and substantiate the role of *panx1a* as a significant molecular hub, which was proposed previously from interactome studies^35^. Moving forward the transcriptome study opens windows of opportunity to study the diverse mechanism dynamically regulating Panx1 channel function, such as elevated extracellular K^+^^36^, or the interaction with purinergic^37,38^, NMDA^39,40^, or α1-adrenergic receptors^41,42^.

The regulation of genes known as primarily located in the pre- and postsynaptic compartments of dopaminergic synapses provide for the first-time evidence that ablation of *Panx1a* alters dopaminergic signaling and that this change was consequential for behavioral outcomes. Cellular effects driven by the loss of *Panx1a* functions reduced the navigational competence of larvae in a dark environment, as well as response to the sudden darkness. This response was reproduced in wild type larvae through pharmacological activation of D1-/D2-like dopaminergic receptors and rescued in mutants by treatment with the D2-like receptor antagonist Haloperidol. The visual-motor behavioral assay (VMR) allowed measuring the responsiveness of zebrafish larvae to changes in light intensities^43^. This responsiveness requires an intact retina^44^, and photoreceptor populations with distinct spectral properties^45^. The advantage of this test compared to the optokinetic reflex test (OKR) is that it allows extracting behavioral responses caused by changes in the retinal ON and OFF pathways. A limitation of the VMR test is that at 6 dpf it does not discriminate between rod and cone responses^46^ since rod photoreceptors are not functional in zebrafish until 15 dpf ^47,48^. Altogether, we propose that the altered behavior of *panx1a*^−/−^ larvae in a dark environment and during light/dark transitions is caused by *Panx1a* modulating visual inputs through D2-like dopaminergic receptors signaling. In line with this proposal is that a recent study reported Panx1 channel activity currents only in the OFF-type retinal ganglion cells (RGCs)^28^, the neurons responsible for detecting light decrement. Beyond the visual system, a recent behavioral study in Panx1 knock out mice showed that loss of Panx1 induces difficulties in motor control and changes of the sleep-wakefulness cycle. The authors suggested deficiencies in adenosine and other signaling pathways were causing the reported deficiency^49^.

Local field potentials were recorded *in vivo* from superficial layers of the optic tectum (OT) to capture changes caused by *Panx1a* modulating visual inputs. Using full-field Light-ON and Light-OFF stimuli, we demonstrated that the neuronal circuitry of 6 dpf larvae responded to light changes with a flexible transition through various brain wave frequencies. This transition is altered in *panx1a*^−/−^ larvae, and reproduced by pharmacological blocking of *Panx1a*, or by treatment with the D1/D2 receptor agonist apomorphine. This result suggests that *Panx1a* affects the role of the optic tectum in modulating the processing of sensory information received from inputs from the retina and that dopaminergic signaling plays a role in this change. This conclusion is consistent with the role of the OT in constructing an image of the physical surroundings, integrating visually acquired information with motor inputs and outputs to initiate appropriate behavioral responses or changes thereof as quantified in this study.

At the level of detail provided here, no simple conclusion can be offered, which explains the full spectrum of differences caused by the ablation of the *panx1a* gene. We speculate that the phenotype is initiated by impaired levels of ATP and adenosine. The results shown here advocate for a role of *Panx1a* in the developmental plasticity of the visual system in a way similar to the plasticity of cortical neurons in Panx1^−/−^ mice^50^. In the retina, from 48 h post-fertilization, RGCs begin to connect with the neuropil of the optic tectum and by 3 dpf retinal stratification is well established^51^. At 3 dpf the optokinetic response is already established, by 5 dpf larvae are capable of tracking and capturing prey, and by 8 dpf the optic tectum is structurally and functionally relatively mature^52^. The size of the visual receptive fields increases from 4 dpf to 6 dpf and then refines to the same size as that at 4 dpf by 8–9 dpf^53^. During this critical developmental period, excitatory components start dominating while GABAergic responses most likely switch from depolarizing to hyperpolarizing currents, making functional pruning of feedforward inputs most likely the most critical factor in receptive field refinement. Input to the optic tectum derives from the retina, striatum, or hypothalamus^54,55^. The superficial layers recorded from in this study receive input from the retina^55^. They are distinct from dopaminergic input from the striatum and the hypothalamus connecting to deep layers of the optic tectum. In the retina, dopamine has long been known to play important modulatory roles in the vertebrate visual system^56–59^. *Panx1a* in the zebrafish retina is located in HCs-cones synaptic complexes^20^, in proximity to TH expressing neurons^19^, where different neurotransmitters like ATP and dopamine act to fine-tune the conductance between cone and HCs. In the darkness, a steady inflow of cations keeps photoreceptors relatively depolarized. Glutamate is released continuously from cones in the synaptic space between cones and HCs. Dopamine increases the conductance of the glutamatergic synapse between cones and HCs through a D1R-mediated mechanism^60^, leading to depolarization of HCs^57,61^. Noteworthy, when HCs are depolarized, in the dark, the Panx1 channel is maximally active and releases ATP into the synaptic cleft, by means of which it participates in sending inhibitory feedback to cones^22,23^. The negative feedback from HCs to cone photoreceptors generates the center/surround organization of bipolar cell receptive fields and is crucial for visual phenomena like contrast enhancement and redundancy reduction^23^.

The vision centric investigation of molecular, electrophysiological, and behavioral properties of *panx1a*^−/−^ zebrafish larvae has uncovered a novel association between *Panx1a* in the integration of sensory-motor behavior through modulation of dopaminergic signaling. We acknowledge that beyond the scope of this study, *Panx1a* could also play other roles in the nervous system, perhaps in neural plasticity transcending a range of spatio-temporal scales which serve non-sensory functions in the motor system, or cognitive functions, such as affecting learning, and memory in mice^9^. The panx1a^−/−^ model will provide a versatile platform for future investigation of these knowledge gaps using genetic, pharmacological, and behavioral phenotyping.

## Methods

### Fish Husbandry and embryo collection

Zebrafish (*Danio rerio*) of strain Tupfel long fin (TL) were used to generate the *panx1a*^−/−^ mutant line^62^. The fish were maintained in a recirculation system (Aquaneering Inc., San Diego, CA) at 28 °C on a 14 hours light/ 10 hours dark cycle. Experiments and procedures with animals were performed at York Universities zebrafish vivarium, according to the CACC guidelines of the Canadian Council for Animal Care (CCAC) after approval of the protocol by the Animal Care Committee (ACC) (GZ#2014-19 (R3). The number of experiments, including zebrafish larvae, was kept to the necessary minimum. The York University Biosafety Committee (YUBC) approved all other experiments (Permit#04-11).

### Generating ***panx1a*** mutant by TALENs

Potential TALENs target sites on *panx1a* (NM_200916.1) were identified using the Mojo Hand software (http://talendesign.org)^63^. Sequence-specific TALEN constructs were assembled using Golden Gate cloning methods^64,65^. A detailed description of the design, preparation, and activity screening of TALENs, as well as microinjection procedure and validation of knockout lines, are provided in the Supplementary Information.

### RNA extraction and RT-qPCR

Total RNAs were extracted from 6 dpf larvae using RNeasy Plus Mini Kit (Qiagen). The iScript Reverse Transcription Supermix (Bio-Rad Laboratories, Mississauga, Canada) was used to reverse transcribe 1 µg of total RNA. The cDNA equivalent of 15 ng total RNA was analyzed in triplicate by quantitative Real Time-PCR using the SsoAdvanced SybrGreen PCR mix (Bio-Rad). All experiments included a melt curve analysis of PCR amplicons generated in each reaction. Raw cycle threshold values (Ct-values) were exported from the CFX Manager Software (Bio-Rad, Canada), and the relative gene expression was calculated using the Relative Expression Software Tool (REST-2009)^66^. Gene information and primer sequences are listed in Supplementary Table S4.

### Transcriptome analysis

The RNA library preparation was performed following the NEB NEBNext Ultra II Directional RNA Library Preparation protocol (New England Biolabs Inc., Ipswich, MA, USA). RNA libraries were loaded on a Bioanalyzer 2100 DNA High Sensitivity chip (Agilent Technologies) to check for size, quantified by qPCR using the Kapa Library Quantification Illumina/ABI Prism Kit protocol (KAPA Biosystems, Wilmington, MA, USA). Pooled libraries were paired-end sequenced on a High Throughput Run Mode flow cell with the V4 sequencing chemistry on an Illumina HiSeq. 2500 platform (Illumina, Inc., San Diego, CA) following Illumina’s recommended protocol to generate paired-end reads of 126-bases in length. The post-sequencing processing to final read counts, normalization, and differential gene expression analysis used multiple software packages, including a two-condition differential expression analysis using the edgeR R-package, v.3.8.6 (http://www.bioconductor.org/packages/release/bioc/html/edgeR.html)^67,68^.

### Behavioral assays

The Zebrabox behavior system and the ZebraLab analytical suite was used for automated observation and video tracking of 6 dpf zebrafish larvae in 48-well plates (ViewPoint Life Technology, Lyon, France; http://www.viewpoint.fr). The visual-motor response (VMR) assay was based on the configurations established elsewhere^43^. Typically, the activities of 48 larvae were recorded simultaneously. Data were collected from video frames at 1-second intervals. For VMR, we analyzed the period from 1 min before the light change to 1 min after the light change (−60-0 sec; 0–1 sec; 1–60 sec) to capture the larval response to abrupt light change. Both assays are described in detail in the online Supplementary Information. All experiments were performed between 12:30 to 4 pm, as larvae (6 dpf) activity was previously reported to reach a stable level by early afternoon^69^.

### *In-vivo* electrophysiology

Published procedures were used to prepare anesthetized zebrafish larvae (6 dpf) for *in vivo* electrophysiology^70^. Microelectrodes were back loaded with 2 M NaCl, and local field potentials were recorded using an Axiocamp 700B amplifier (Axon Instruments, San Jose, CA, USA). Typically, microelectrodes had a resistance of 2–7 M. They were placed into the optic tectum. Recordings were low-pass filtered at 1 kHz (-3 dB; eight-pole Bessel), high-pass filtered at 0.1–0.2 Hz, digitized 10 kHz using a Digidata 1550 A A/D interface, and stored on a PC computer running pClamp11 software (Axon). The basal activity was recorded for 10 minutes under LIGHT-on Light-ON conditions. The basal activity during LIGHT-off Light-OFF was recorded for another 10 minutes. In some experiment’s zebrafish larvae were pre-incubated for 1 hr with 100 µM Probenecid. During recordings, probenecid was present in the bath solution.

### Pharmacology

All the chemicals were purchased from Sigma-Aldrich (Mississauga, Canada): R-(−)-apomorphine hydrochloride hemihydrate (cat#A4393), Probenecid (cat#P8761), Pancuromium bromide (cat#P1918), Ethyl3-aminobenzoate methanesulfonate (MS-222; A5040), R(+)-SCH-23390 hydrochloride (cat#D054), and Haloperidol (cat#H1512).

### Statistics

Unless otherwise stated, all statistical analyses were performed in R software version 3.4.0 (http://www.r-project.org), and results are represented as the mean ± standard error of the mean (SEM) of 3 repeated experiments. A *p*-value < 0.05 was considered statistically significant. The average values for protein assays, cell count, electrophysiology data, traveled distance and velocity, were compared between two groups using Student’s *t*-test (with equal variance) or Welch’s *t-*test (without equal variance) as indicated. Details of procedures and software packages used can be found in the Supplementary Information section.

## Supplementary information

Supplemental information.

## Data Availability

RNA-seq expression data have been deposited at the NCBI Gene Expression Omnibus (GEO) database (GSE147068).
