# Supplementary material for: Visuomotor deficiency in panx1a knockout zebrafish is linked to dopaminergic signaling
Source: Sci Rep. 2020 Jun 12;10:9538. doi: 10.1038/s41598-020-66378-y (PMC7293225; doi:10.1038/s41598-020-66378-y)
Supplement: Supplementary file 1 — Supplemental information. [file 41598_2020_66378_MOESM1_ESM.docx]

## **Visuomotor deficiency in *panx1a* knockout zebrafish is linked to dopaminergic signaling**

## Nickie Safarian, Paige Whyte-Fagundes, Christiane Zoidl, Jörg Grigull, Georg Zoidl^*^

## **Affiliations**

## Department of Biology, York University; Toronto, Ontario, M3J1P3; Canada

## Nickie Safarian

## Paige Whyte-Fagundes

## Christiane Zoidl

## Georg Zoidl

## Department of Mathematics and Statistics, York University; Toronto, Ontario, M3J1P3; Canada

## Jörg Grigull

## Center of Vision Research, York University; Toronto, Ontario, M3J1P3; Canada

## Nickie Safarian

## Paige Whyte-Fagundes

## Georg Zoidl

**Corresponding author**

Correspondence to [gzoidl@yorku.ca](mailto:gzoidl@yorku.ca)

## **Supplementary Information**

**TALEN Design -** Potential TALENs target sites on *panx1a* **(**NM_200916.1) were identified using Mojo Hand software (<http://talendesign.org>) ^1,2^. The following criteria were used for TALEN design: TALENs target sites were 15-17 bases long with an initial 5’ T nucleotide to the TALE domain. The spacer length was restricted to 15-16 base pairs. Target sites with a unique restriction enzyme sequence located in the middle of the spacer sequence were selected to simplify screening for insertion-deletion (indel) mutations. The specificity of selected TALENs target sequences was determined using the BLAST interface build into the Mojo Hand software.

**TALEN Constructs -** The TALEN constructs were synthesized in Dr. Stephen Ekker’s lab (Mayo Clinic Cancer Center, Rochester, MN). Briefly, TALEN assemblies of the RVD-containing repeats were conducted using the Golden Gate approach ^3^. Once assembled, the TALE repeats were cloned in the pT3TS-GoldyTALEN expression vector ^4,5^. TALEN expression vectors were linearized with the *Sac*I restriction endonuclease (ThermoFisher Scientific, Canada) for 15 min at 37°C, and used as templates for *in vitro* transcription. Capped cRNAs were synthesized from TALEN pairs mixed 1:1 using the mMESSAGE mMACHINE T3 Transcription kit (Life Technologies, Canada). The mixture of the two TALEN cRNAs was purified using the Oligotex mRNA Mini Kit (Qiagen Inc., Toronto, Canada). TALEN cRNAs were diluted in DNase/RNase-free water (Life Technologies) to the final concentration of 1 µg/μL and stored at −80 °C before microinjection.

**TALEN Activity Screening -** One-cell stage zebrafish embryos were injected with TALEN cRNAs pair at doses ranging from 30-100 pg/nl. The toxicity of the injected cRNAs was determined at 24 hours post fertilization (1dpf) by calculating the proportion of healthy, dead, and malformed embryos at each dose. The condition resulting in more than 50% post-injection survival was selected for further injections. Genomic DNA (gDNA) was extracted from groups of 10 injected embryos at four days post-fertilization (4dpf) To examine the TALEN mutagenesis efficiency. Briefly, the individual larva was incubated in 100mM NaOH at 95$\boldsymbol{℃}$ for 15 min. After cooling to room temperature, the one-tenth volume of 1 M Tris (pH8.0) was added to the extracts to neutralize the NaOH ^6^. Finally, 1 volume TE buffer pH8.0 was added, and gDNAs were stored at -20$\boldsymbol{℃}$. A small indel mutation screen used PCR followed by *AfeI* restriction enzyme (RE) digest. Indel mutations were confirmed by sequencing (Eurofins Genomics LLC, KY, USA) of gel-purified PCR products cloned into the pJet1.2 cloning vector (Life Technologies.

**Generation of *panx1a^-/-^* Zebrafish –** Adult *panx1a* knock-out zebrafish (F0) were anesthetized in pH-buffered 0.2mg/ml ethyl3-aminobenzoate methane sulfonate solution (MS-222, Sigma-Aldrich). The caudal fin (2 mm of the end) was removed using dissecting scissors (WPI Inc., FL, USA) and placed into 1.5 ml collecting tubes. The fin gDNA was isolated and screened for indel mutations as described ^4^. Adult F0 zebrafish with a mutation in the *panx1a* gene were out-crossed to wild-type (WT) TL zebrafish and, F1 offspring were analyzed by PCR and *AfeI* digestion to verify germline transmission of mutations. Heterozygous (*panx1a*^+/-^) F1 mutants were in-crossed to establish homozygous F2 *panx1a*^-/-^ mutants. All experiments described were performed with progenies of the *panx1a* ^-/-^ F3 generation.

**Western blot and confocal microscopy -** Neuroblastoma 2a (Neuro2a; ATCC-CCL-131) cells were maintained using standard growth conditions and used for *Panx1a* expression and localization studies ^7^. Primary antibodies were anti-tyrosine hydroxylase (*th*, 1:500; Sigma-Aldrich cat# SAB2701683, Canada) and mouse anti-β-actin (*actb*, 1:1,000 dilution, cat# clone AC-40, Sigma-Aldrich). The IRDye 800cw or 680RD secondary antibodies (LI-COR Biosciences, St. Lincoln, NE, USA) were diluted 1:15,000. Signals were detected using an Odyssey CLx imaging system (LI-COR Biosciences). Confocal images of transfected cells were processed with LSM-ZEN software (Zeiss LSM700 system; Carl Zeiss MicroImaging, Oberkochen, Germany) using a Plan-Apochromat 63x/1.40 Oil DIC M27 oil immersion lens.

**Immunohistochemistry –** Adult zebrafish were humanely euthanized in MS-222 solution (0.02% w/v, Sigma-Aldrich). Eyes were removed and fixed in 4% paraformaldehyde (PFA) in 1xPBS overnight at 4^o^C, followed by cryoprotection in 30% sucrose in 1xPBS. After embedding in Tissue-Tek O.C.T compound 10µm sections were cut on a cryotome (Thermofisher). Samples were washed three times for 5 min with 1xPBS containing 0.1% Tween-20 (PBST) at RT. Unspecific binding sites were blocked with freshly prepared 5% normal goat serum (NGS, Sigma-Aldrich) in PBST for 4hr at 4$℃$. Following blocking, samples were incubated with primary antibody (1:100, affinity-purified rabbit anti-*panx1a* antibody, code#ZOI-A2, Davids Biotechnologie GmbH; 1:500, anti-PSD-95, #clone 7E3-1B8, Sigma-Aldrich) overnight at 4℃. Subsequent washes with PBST were for one hour at 4$℃$. Alexa 488 and Alexa 546 goat anti-rabbit/mouse secondary antibodies (1:3,000 in 1% NGS PBST, Life Technologies) was applied for one hr at RT$℃.$ After 3 washed with PBST followed by one wash with PBS, specimens were mounted in on microscope slides using ProLong Antifade with DAPI (Thermofisher). Confocal images were collected using LSM-ZEN2 software (Zeiss LSM700 system; Carl Zeiss MicroImaging, Oberkochen, Germany) with a Plan-Apochromat 63x/1.3 oil DIC M27 objective using the identical settings.

**Whole-Mount Immunohistochemistry –** Larvae at 3dpf were chosen over 6dpf larvae because of more efficient antibody penetration and low pigmentation. At this stage all layers of the retina are fully developed ^8^. The larvae were euthanized in MS-222 solution (0.02% w/v, Sigma-Aldrich) and fixed in 4% paraformaldehyde (PFA) in 1xPBS overnight at 4$℃$. Samples were washed three times for 5 min with 1xPBS containing 0.1% Tween-20 (PBST) before a permeabilization step with pre-chilled acetone for 20 min at -20$℃$. Unspecific binding sites were blocked with freshly prepared 10% normal goat serum (NGS, Sigma-Aldrich) in PBST for 4hr at 4$℃$. Following blocking, samples were incubated with primary antibody (1:200, affinity-purified rabbit anti-TH antibody, Sigma-Aldrich cat# SAB2701683) for 2-3 days at 4℃. Subsequent washes with PBST were for 5 hours at 4$℃$. Alexa 488 goat anti-rabbit secondary antibody (1:500 in 1% NGS PBST, Life Technologies) was applied for 48 h at 4$℃.$ Specimens were infiltrated with 75% glycerol in 1xPBS, mounted on microscope slides using 0.8% low melt point agarose (BioShop, Canada). Images were acquired using a Plan-Apochromat 20X/0.8 objective and were processed using Zeiss LSM-ZEN software and the Fiji program ^9^. The brightness and contrast of the images were adjusted using the Fiji program. Whole retinas were positioned laterally, or flat, and Z-stack images taken. The data were stored and used for off‐line analysis. When indicated, z-stacks were deconvoluted using the ImageJ plugins Iterative Deconvolve3D and Diffraction PSF3D ^10^. The large cell bodies of tyrosine hydroxylase stained cells ^11-13^ were counted manually by two individuals. Data were expressed as mean ± SD. Non-injected larvae or excluding the primary antibody served as controls.

**Behavioral Assays** – Larvae were tested at 6dpf using a Zebrabox behavior recording system (ViewPoint Life Technology, Lyon, France; <http://www.viewpoint.fr>), and the Zebralab software (ViewPoint Life Technology, Lyon, France; <http://www.viewpoint.fr>). Tracking videos were recorded at 30 frames per second (fps) under infrared light illumination using a Point Grey Research Dragonfly2 DR2-HIBW. A lightbox provided infrared (for Light-OFF recording) or visible light (for Light-ON recording). All experiments were performed at 28^o^C. At least three independent experiments were performed for each assay type.

**Freely swimming behavior assay** – Larval swimming activity under constant Light-ON/OFF conditions was tested using 48-well plates. Larvae were adapted to the environment for 3hrs before recording begins. For tracking in light, larvae were adapted to ambient light (30% of final output intensity). For tracking in the dark, larvae were acclimatized to darkness (0% of final output intensity) for 3hrs. Next, locomotor behavior was tracked for 60 min. For analysis of locomotion, three thresholds were defined: slow (<2mm), medium (2-20mm), and fast (>20mm). The mean traveled distance (mm) and velocity (mm/sec) in two swim speeds (medium and fast) was used for statistical analysis.

**The visual-motor response (VMR) assay** – The VMR assay was implemented as reported ^14^. Larvae in 48-well plates were adapted to darkness (Light OFF) for 3hrs. After dark adaptation, baseline locomotion was recorded. Then, three trials of alternating light onset (Light ON) and light offset (Light OFF) periods with each period lasting 30 minutes, for a total of 180 min, followed. The Light-ON stimulus was set to 100%, and the Light-OFF stimulus was set at 0% of the final output intensity. In the Quantization mode of the Zebralab software threshold settings for activity detection in successive video frames were defined as no movement (<6), medium (6-20), activity burst (>20). Activity data were collected per second, with periods selected for analysis outlined in results.

**Principal Component Analysis -** VMR data represent seven variables: the counts and the duration of time larvae spend performing each of the three predefined swimming parameters (i.e., freeze, scoot, and burst) plus the total activity duration (TAD). In order to unambiguously evaluate the nature of behavioral responses to visual stimuli, the multiple components were disentangled by a principal component analysis (PCA) using the "FactoMineR” and “factoextra" packages. First, the PCA scores were Varimax rotated in accord with the standard PCA approach ^12^. The orthogonal configuration of varimax rotation guarantees the different components are uncorrelated, thus each represents an independent behavioral pattern. The resulting values termed "eigenvalues" signify the amount of variance explained by each of the components. To visualize and compare the size of the eigenvalues we used Scree plots. Only the first two components were retained for further interpretation since their eigenvalues were greater than 1 and they accounted for more than 90% of total variance in the data. Next, the variables contribution percentage to each of the two retained components were computed and plotted. The most informative variable, "TAD", was compared between groups using nonparametric rank-based exact multiple contrast testing and simultaneous confidence intervals (MCTP/ SCI) (*nparcomp* package; <http://www.R-project.org>). Mean TAD values of the entire test period were plotted to show control and mutant larvae activity patterns. The activity data from 1 min before (-60 sec) to 1 min after (+60 sec) light changes were plotted separately to visualize the locomotor activity differences of the two genotypes upon after abrupt light switches. To visualize the effects of different drugs treatments on "frect" and "TAD" of larvae upon light switches, we used line charts. The baseline activity (from 1 min before light switches) were used to compare the amount of change occurring in any of the two mentioned activity features during the first 1 second after light switches.

**Electrophysiology** - Zebrafish larvae (6dpf) were anesthetized using 0.02% MS222 and 0.3mM of Pancuronium bromide (both Sigma-Aldrich). Anesthetized larvae were immobilized in freshly prepared 1.2% low melting temperature agarose. An Olympus dissecting microscope was used to orient larvae in agarose so that the dorsal aspect was exposed to the agarose gel surface. Embedded larvae on the sample holder were placed on the upright stage of an Olympus BX51 fluorescence microscope. 2mL of egg water (pH 7.2-7.4) was applied to the agar topically. Under direct visual guidance, a glass microelectrode (1.2mM OD, approximately 1µM tip diameter, 2-7MΩ) was placed into the optic tectum. Microelectrodes were back loaded with 2M NaCl. Electrical activity was recorded using a Multiclamp 700B amplifier (Axon Instruments, San Jose, CA, USA). Voltage recordings were low-pass filtered at 1kHz (-3 dB; eight-pole Bessel), high-pass filtered at 0.1 Hz, digitized 10 kHz using a Digidata 1550A A/D interface, and stored on a PC computer running pClamp11 software (all Axon Instruments). The basal activity was recorded for 10 minutes under Light-ON conditions (1000 lux), during which images of the electrode placement were taken. Then, basal activity was recorded for another 10 minutes with Light-OFF. For analysis purposes, activity during dark conditions was taken from a window in the last 2 minutes of the recording to ensure appropriate adaptation to the change in light stimulus. For drug exposure, zebrafish larvae were pre-incubated in solutions containing 100µM Probenecid for 1 hour. Probenecid was also added to both the agar and recording solution in order to keep steady concentrations.

**Supplementary Figure**

**Supplementary Fig. S1.** Whole-mount immunohistochemistry of 3dpf a) *panx1a^+/+^* and b) *panx1a^-/-^* larva. *Panx1a* immunoreactivity was identified in a region associated with horizontal cells (HCL) adjacent to the photoreceptor cell layer (PCL) (top left). In sagittal sections arrows indicate the position of the ganglion cell layer (GCL), inner nuclear layer (INL), and outer nuclear layer (ONL). Reduced *Panx1a* immunoreactivity was found in the outer retinal layer of 3dpf *panx1a^-/-^* larva only when brightness of the image was enhanced. The significant fluorescence observed in the sclera-choroid region was attributed to *Panx1* expression in this vascular-rich zone, or the cross-reactivity of the antibody, most likely with collagen fibers produced by endothelial cells. Note that images in a) and b) were collapsed from three 0.64-µm-thick optical sections. Scale bar: 25µm c) Sagittal section of an age-matched eye of a *panx1a^+/+^* larva. Tyrosine hydroxylase-immunoreactivity (*th*) was detectable across multiple layers. The arrow indicates the body of a *th*-immunoreactive cell in the inner nuclear layer. Scale bar = 30µm. Horizontal sections in d) and e) derive from a z-stack representing a *panx1a^-/-^* eye. Arrows point at the position of *th*-positive cell bodies located in the inner nuclear layer. Scale bar top and bottom = 20µm. In c) to e) *th*-immunoreactivity was speckled across multiple layers in a similar way as described for *th*-positive cells in higher vertebrates ^13^. Note that all images in c) to e) represent single 0.83-µm-thick optical sections after deconvolution.

**
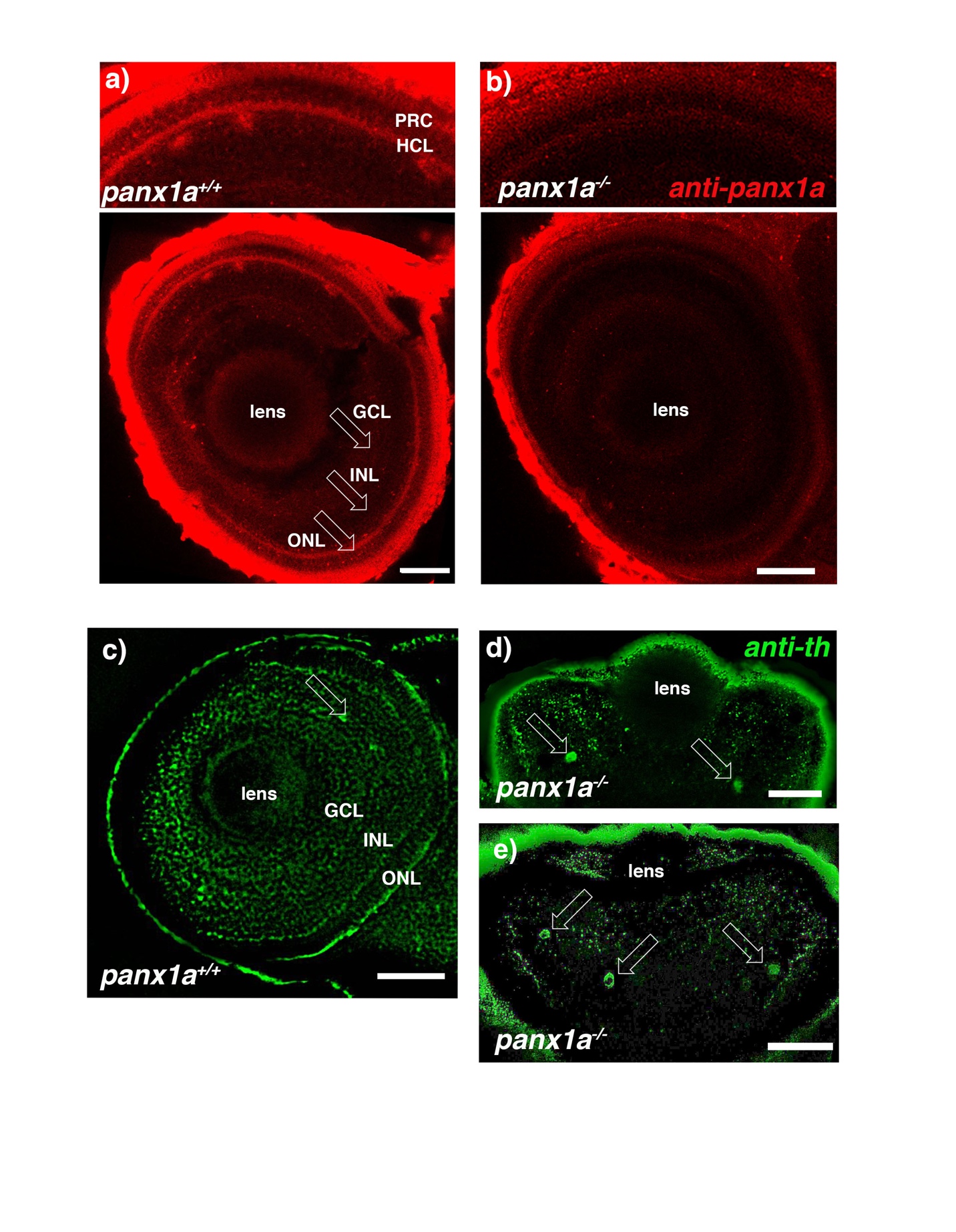
**

**Supplementary Fig. S2. *Apomorphine Dose-Response Test***

Apomorphine administration had dose-dependent effects on locomotion in Light-ON (a) and Light-OFF conditions (b). a) Doses in the 0.1µM to 10 µM range significantly decreased larval activity in light (p-value<0.001). Doses in the 20µM and 50 µM range did not significantly alter locomotion. b) In the dark, significant changes in activity levels were detected after treatment with a low dose (1 µM; p-value=0.02) and a high dose (50 µM; p-value=0.043). TAD data are presented as mean activity ± SEM, n = 24/dose (*p ≤ 0.05; ***p≤ 0.001).

*
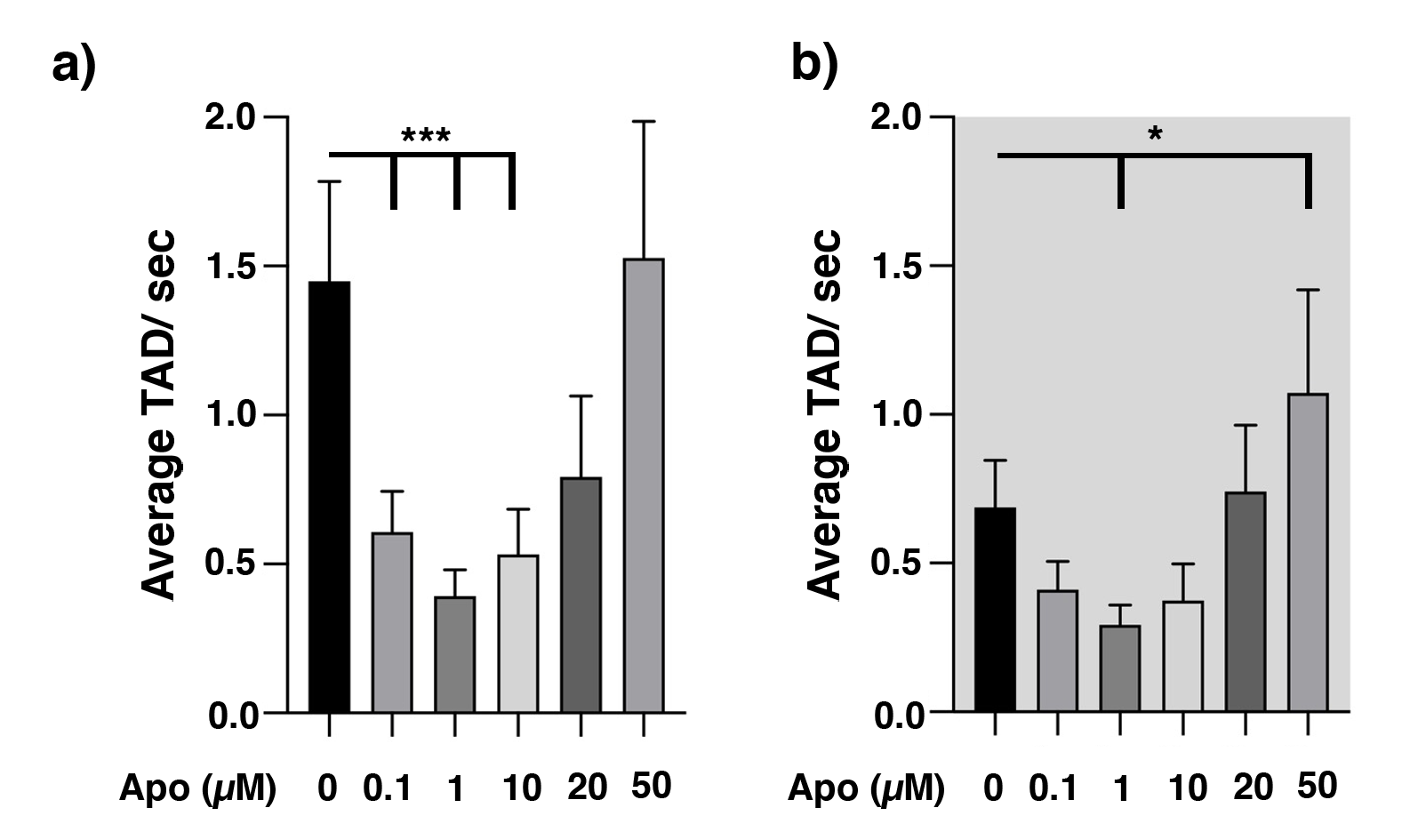
*

**Supplementary Tables**

**Supplementary Table S1a.** *GO annotation for upregulated genes.*

| **Reactome pathways** | ***Danio rerio* (REF)**  **#** | **upload**  **#** | **Fold Enrichment** | ***p*-value** |
| --- | --- | --- | --- | --- |
| **Unclassified** | 16859 | 475 | 0.86 | 0 |
| **The phototransduction cascade** | 44 | 16 | 11.13 | 0.000000054 |
| **Inactivation, recovery and regulation of the phototransduction cascade** | 42 | 15 | 10.93 | 0.000000273 |
| **Visual phototransduction** | 92 | 18 | 5.99 | 0.0000166 |
| **Processing of Capped Intron-Containing Pre-mRNA** | 242 | 26 | 3.29 | 0.000716 |
| **mRNA Splicing** | 194 | 22 | 3.47 | 0.0024 |
| **Activation of the phototransduction cascade** | 14 | 7 | 15.31 | 0.00371 |
| **mRNA Splicing - Major Pathway** | 185 | 20 | 3.31 | 0.0135 |
| **Gene expression (Transcription)** | 803 | 51 | 1.94 | 0.0232 |
| **G alpha (i) signalling events** | 341 | 28 | 2.51 | 0.0412 |

**Supplementary Table S1b.** *GO annotation for downregulated genes.*

| **Reactome pathways** | ***Danio rerio* (REF)** | **upload**  **#** | **Fold Enrichment** | ***p*-value** |
| --- | --- | --- | --- | --- |
| **Unclassified** | 16859 | 902 | 0.8 | 0 |
| **Developmental Biology** | 395 | 73 | 2.78 | 7.25E-10 |
| **Metabolism** | 1825 | 205 | 1.69 | 4.95E-09 |
| **Axon guidance** | 303 | 52 | 2.58 | 0.0000174 |
| **Neutrophil degranulation** | 497 | 71 | 2.15 | 0.000048 |
| **MET promotes cell motility** | 54 | 19 | 5.29 | 0.00011 |
| **MET activates PTK2 signaling** | 37 | 16 | 6.5 | 0.000123 |
| **Signaling by MET** | 98 | 25 | 3.83 | 0.00021 |
| **Hemostasis** | 500 | 69 | 2.07 | 0.000229 |
| **Signal Transduction** | 2196 | 212 | 1.45 | 0.000332 |
| **Signaling by Receptor Tyrosine Kinases** | 449 | 62 | 2.07 | 0.001 |
| **Innate Immune System** | 879 | 100 | 1.71 | 0.00165 |
| **Formation of the cornified envelope** | 39 | 14 | 5.39 | 0.00469 |
| **Keratinization** | 39 | 14 | 5.39 | 0.00469 |
| **Neuronal System** | 434 | 58 | 2.01 | 0.00472 |
| **Immune System** | 1420 | 143 | 1.51 | 0.0051 |
| **Membrane Trafficking** | 518 | 64 | 1.86 | 0.0182 |
| **Vesicle-mediated transport** | 541 | 65 | 1.81 | 0.0291 |
| **CRMPs in Sema3A signaling** | 19 | 9 | 7.12 | 0.0499 |

**Supplementary Table S2.** *Comparison of transcriptional changes of selected transcripts by RNA-seq and RT-qPCR.*

| **Gene** | **RNA-seq**  **Log2 expression** | **p-value** | **FDR** | **RT-qPCR**  **Expression (REST)** | **S.E. (REST)*** | **P-value (REST)*** | **Result** |
| --- | --- | --- | --- | --- | --- | --- | --- |
| ***opn1lw2*** | 0.496 | 0.073 | 0.417 | 1.052 | 0.508 - 2.153 | 0.799 |  |
| ***opn1mw1*** | 0.276 | 0.104 | 0.572 | 1.075 | 0.302 - 2.463 | 0.836 |  |
| ***opn1mw2*** | 1.366 | 0.103 | 0.000 | 1.074 | 0.601 - 1.935 | 0.671 |  |
| ***opn1sw1*** | 0.976 | 2.105 | 0.016 | 4.301 | 1.903 - 12.773 | 0 | UP |
| ***opn1sw2*** | 0.918 | 0.995 | 0.025 | 1.993 | 0.722 - 4.186 | 0.001 | UP |
| ***opn4.1*** | 1.672 | 1.006 | 6.23E-05 | 2.009 | 1.216 - 3.579 | 0.001 | UP |
| ***rho*** | 1.066 | 0.861 | 0.010 | 1.816 | 0.674 - 3.811 | 0.008 | UP |
| ***gnat1*** | 0.850 | 1.940 | 0.012 | 3.837 | 2.058 - 8.380 | 0 | UP |
| ***gnat2*** | 1.353 | 2.090 | 0.002 | 4.257 | 2.313 - 8.121 | 0 | UP |
| ***pde6c*** | 1.418 | 1.196 | 0.001 | 2.291 | 1.236 - 5.034 | 0 | UP |
| ***pde6ha*** | 1.225 | 2.646 | 0.007 | 6.26 | 3.374 - 11.990 | 0 | UP |
| ***guca1c*** | 2.791 | 1.375 | 1.54E-06 | 2.593 | 1.017 - 5.043 | 0 | UP |
| ***gucy2d*** | 1.094 | 2.179 | 0.018 | 4.529 | 2.527 - 8.475 | 0 | UP |
| ***gna11b*** | 1.447 | 1.189 | 6.69E-05 | 2.28 | 1.180 - 4.497 | 0 | UP |
| ***grk7b*** | 1.587 | 1.006 | 6.96E-05 | 2.009 | 1.216 - 3.579 | 0.001 | UP |
| ***p2rx1*** | -1.941 | 1.6E-04 | 0.007 | 0.682 | 0.382 - 1.122 | 0.019 | DOWN |
| ***p2rx5*** | -1.602 | 0.9E-04 | 0.008 | 0.379 | 0.098 - 1.251 | 0.009 | DOWN |
| ***p2rx7*** | -1.899 | 1.51E-10 | 3.86E-08 | 0.278 | 0.031 - 1.179 | 0.019 | DOWN |
| ***adora1b*** | -1.256 | 0.9E-04 | 0.023 | 0.454 | 0.201 - 0.994 | 0.001 | DOWN |
| ***cacna1da*** | -0.898 | 0.9E-04 | 0.008 | 0.124 | 0.040 - 0.281 | 0 | DOWN |
| ***kcnc1a*** | -1.497 | 8.72E-08 | 7.55E-06 | 0.073 | 0.015 - 0.323 | 0 | DOWN |
| ***kcnc1b*** | -1.680 | 2.29E-07 | 1.58E-05 | 0.537 | 0.252 - 1.047 | 0.005 | DOWN |
| ***kcna2b*** | -1.815 | 2.48E-06 | 1.0E-4 | 0.413 | 0.205 - 0.783 | 0.001 | DOWN |
| ***kcnh4a*** | -2.035 | 4.01E-06 | 1.0E-4 | 0.483 | 0.212 - 1.177 | 0.006 | DOWN |
| ***trpv4*** | -2.065 | 0.001147 | 0.009 | 0.216 | 0.060 - 0.659 | 0 | DOWN |
| ***trpm6*** | -1.237 | 0.00043 | 0.005 | 0.124 | 0.040 - 0.281 | 0 | DOWN |

*Values were calculated using the Relative Expression Software Tool (REST) ^15^.

**Supplementary Table S3.** *RT-qPCR results for transcripts associated with the dopaminergic pathway.*

| **Gene** | **RT-qPCR**  **Expression (REST)** | **S.E. (REST)** | **P-value (REST)** | **Result** |
| --- | --- | --- | --- | --- |
| ***drd1a*** | 1.364 | 0.812 - 2.237 | 0.045 | UP |
| ***drd1b*** | 1.676 | 0.696 - 3.555 | 0.027 |  |
| ***drd2a*** | 2.328 | 1.294 - 3.938 | 0.000 | UP |
| ***drd2b*** | 1.635 | 0.677 - 3.535 | 0.028 | UP |
| ***drd2c*** | 1.294 | 0.796 - 2.110 | 0.096 | UP |
| ***drd3*** | 3.397 | 1.359 - 9.037 | 0.001 | UP |
| ***drd4a*** | 3.336 | 1.519 - 7.193 | 0.000 |  |
| ***drd4b*** | 1.216 | 0.765 - 1.990 | 0.190 | UP |
| ***drd5a*** | 2.060 | 0.815 - 4.818 | 0.003 | UP |
| ***th*** | 6.082 | 2.919 - 13.157 | 0.000 | UP |
| ***ddc*** | 1.566 | 0.732 - 3.248 | 0.060 | UP |
| ***mao*** | 1.660 | 0.805 - 3.589 | 0.026 |  |
| ***comta*** | 0.991 | 0.493 - 1.948 | 0.970 |  |
| ***slc6a3*** | 2.958 | 1.122 - 6.871 | 0.001 | UP |
| ***slc18a2*** | 2.119 | 1.209 - 4.015 | 0.000 | UP |

**Supplementary Table S4**. *Summary of Genes and Primers.*

| **Gene** | **Gene-ID** | **Function** | **Forward Primer (5‘ – 3‘)** | **Reverse Primer (5‘ – 3‘)** | **Application** |
| --- | --- | --- | --- | --- | --- |
| ***opn1lw2*** | NM_001002443 | G-protein coupled receptor | ccaacagcaataacacaaggg | gcgacaaccacaaagaacatc | RT-qPCR |
| ***opn1mw1*** | NM_131253 | G-protein coupled receptor | ggctgtgtaatggagggattc | atggtttgcggagaatttgaag | RT-qPCR |
| ***opn1mw2*** | NM_182891 | G-protein coupled receptor | gctttcgctggaacaattatgg | acaagagaccaaagagcaacc | RT-qPCR |
| ***opn1sw1*** | NM_131319 | G-protein coupled receptor | tcattttctcctactcacagctc | cacaaaagagccaaccatcac | RT-qPCR |
| ***opn1sw2*** | NM_131192 | G-protein coupled receptor | ggttcctttcagcaccattg | agaagccgaacaccattacc | RT-qPCR |
| ***opn4.1*** | NM_178289 | G-protein coupled receptor | gtgattggaaatgcactggtg | ctgtgtaaactggctgcaaag | RT-qPCR |
| ***rho*** | NM_131084 | G-protein coupled receptor | agtcctgcccagacatctag | gtactgtgggtattcgtatggg | RT-qPCR |
| ***gnat1*** | NM_131868 | heterotrimeric G-protein | cgtcaagtttgtgttcgatgc | gaggaaacgagctacaaggag | RT-qPCR |
| ***gnat2*** | NM_131869 | heterotrimeric G-protein | caaacctgactaccttcccac | tcttcctctcggacctctg | RT-qPCR |
| ***pde6c*** | NM_200871 | cGMP 3',5'- cyclic phosphodiesterase | CATATTTGCCACAGTCATCGC | AGAGCCTTGTGAAACTGACG | RT-qPCR |
| ***pde6ha*** | NM_001305554 | cGMP 3',5'- cyclic phosphodiesterase | aagttcaagcagaggaccac | tccatgtctccaaacgcttc | RT-qPCR |
| ***guca1c*** | NM_194393 | guanylate cyclase activator | tttcgtggagtacatcgctg | ggtctccatttcatctctgtcg | RT-qPCR |
| ***gucy2d*** | NM_131866 | guanylate cyclase | agaaggaaactgaatgacgagag | cccagtcaccctcaaatacag | RT-qPCR |
| ***gna11b*** | NM_001007773 | heterotrimeric G-protein | tttgtggatctgaaccctgac | aagcggatgttctctgtgtc | RT-qPCR |
| ***grk7b*** | NM_001033090 | non-receptor serine/threonine protein kinase | acgagaaggagatcaagaaacg | actgacggaaacatgctctc | RT-qPCR |
| ***p2rx1*** | NM_198982 | P2X purinoreceptor | CCCAGTTTGATGTAGTCAGGAG | AAACTGAACCCGTATCGCTC | RT-qPCR |
| ***p2rx5*** | NM_194413 | P2X purinoreceptor | AGTAAATGGAAAGGCTGGGAG | CTCTCGGTAAAAGGAGCTCTTG | RT-qPCR |
| ***p2rx7*** | NM_198984 | P2X purinoreceptor | CCTTACAGGAGCAACTGTGC | AAAGCGTTCTCCAGGACAGA | RT-qPCR |
| ***adora1b*** | NM_001128584 | adenosine receptor subunit | GGAACAATTTACACAGCCTGC | ACGAGCATGAAAAGCAGAGG | RT-qPCR |
| ***cacna1da*** | NM_203484 | voltage-dependent l-type calcium channel subunit | ggatgagaaggataatgccgag | gggtttgtgttgctgaagatg | RT-qPCR |
| ***kcnc1a*** | NM_001128725 | potassium voltage-gated channel subfamily c | tgtaagtctgccatgaactcg | ttcactttgggatctgctctg | RT-qPCR |
| ***kcnc1b*** | NM_001195197 | potassium voltage-gated channel subfamily c | ctgtcctcacatcgaccaag | cataacccttcctgactcttcc | RT-qPCR |
| ***kcna2b*** | NM_001362628 | \| potassium voltage-gated  channel subfamily a \| \| --- \| \|  \| | CATTGGAGGCAAAATCGTGG | TGTTCCTCTCCCTCTGTCTC | RT-qPCR |
| ***kcnh4a*** | NM_001322433 | potassium voltage-gated channel, subfamily h | cacaacctcacctacaacctc | gtctcctgcctaaatcctgc | RT-qPCR |
| ***trpv4*** | NM_001042730 | transient receptor potential cation channel subfamily v | gagatcccaacttccccatg | ttctgcttctggtcgttctg | RT-qPCR |
| ***trpm6*** | NM_001251831 | transient receptor potential cation channel subfamily m | tgtcgtgctaagtatgtgcg | gaagttatctgtccctccgtg | RT-qPCR |
| ***drd1a*** | XM_017359120 | G-protein coupled receptor | ctcatctccttcatcccagtg | atatgttcggttgaggctgg | RT-qPCR |
| ***drd1b*** | NM_001135976 | G-protein coupled receptor | acgctgtccatccttatctc | tgtccgattaaggctggag | RT-qPCR |
| ***drd2a*** | NM_183068 | G-protein coupled receptor | atcgggatgggtgcatttc | tggtactccggaaaagacg | RT-qPCR |
| ***drd2b*** | NM_197936 | G-protein coupled receptor | ACCTCCAAGTCCCAATCATG | GTTCGGGTTTTGCCATTAGG | RT-qPCR |
| ***drd2c*** | AY333792 | G-protein coupled receptor | acctccaagtcccaatcatg | gttcgggttttgccattagg | RT-qPCR |
| ***drd3*** | NM_183067 | G-protein coupled receptor | atcagtatcgacaggtatacagc | ccaaacagtagagggcagg | RT-qPCR |
| ***drd4a*** | NM_001012616 | G-protein coupled receptor | cttaccgctgtttgtgtatgc | atgaacctgtctatgctgatgg | RT-qPCR |
| **drd4b** | NM_001012618 | G-protein coupled receptor | ttacccctgtttgtctatgctg | ggctataaacctgtccacactg | RT-qPCR |
| **drd5a** | XM_003199767 | G-protein coupled receptor | ACCAACTCTTCGCTCAACC | TCGTTGCTGATGTTGACCG | RT-qPCR |
| **th** | NM_001001829 | monooxygenase | ttgtgtccgagagctttgag | aagcattctggatcttggagg | RT-qPCR |
| **ddc** | NM_213342 | decarboxylase | CCGCAAGCATGTAGGACTG | GCTGTTGATCCTCTTCAGCAG | RT-qPCR |
| **slc6a3 (DAT)** | NM_131755 | transporter | gaatcgacagtgctatgggag | cagatgagcgagatgaggaag | RT-qPCR |
| ***slc18a2 (VMAT2)*** | NM_001256225 | transporter | agctccttttcttatcctggc | tgcaatgaggatgtatgggtc | RT-qPCR |
| ***comta*** | NM_001030157 | methyltransferase | tgaccactattgcagacacag | gatgcgaacagtagagtatccg | RT-qPCR |
| ***mao*** | NM_212827 | oxidase | cggctttgtggtttctttgg | gcttcactcgatcacccag | RT-qPCR |
| ***panx1a*** | NM_200916 | pannexin | TGGATACAAACAGCTCTGTGATAG | ATCCTTTAGAGTAGCGCTTGG | RT-qPCR |
| ***panx1b*** | NM_001100030 | pannexin | AAGTATAAAGGCGTGCGGCTGG | TGATCTGAGTACCCACGGAGAC | RT-qPCR |
| ***panx2*** | NM_001256641 | pannexin | TCGACTGAGAGGACAAACCC | TCGCCACCAACTTCACAACTC | RT-qPCR |
| ***panx3*** | XM_001919826 | pannexin | CATACAACCGCTCTGTCCGT | GCTTGCGTTTGGCTCTCTC | RT-qPCR |
| ***panx1a*** | NM_200916 | pannexin | AGCTCTGTGATAGACCTCACTGAGAGC | GATGTAGCTCAGGAGCTGAAAGATGC | genotyping |
| ***actb1*** | NM_131031 | actin, cytoplasmic | tgagcaggagatgggaacc | caacggaaacgctcattgc | Normalization |
| ***actb2*** | NM_181601 | actin, cytoplasmic | gcccctagcacaatgaagatc | gactcatcgtactcctgcttg | Normalization |
| ***tuba1b*** | NM_194388 | tubulin, cytoplasmic | GAGCGTCCTACTTACACCAAC | AGGGAAGTGGATACGAGGATAG | Normalization |
| ***b2m*** | L05383 | major histocompatibility complex antigen | GCCTTCACCCCAGAGAAAGG | GCGGTTGGGATTTACATGTTG | Normalization |
| ***tbp*** | NM_200096 | tata box- binding protein-like protein | cggtggatcctgcgaatta | Tgacaggttatgaagcaaaacaaca | Normalization |
| ***eef1a1l1*** | NM_131263 | eukaryotic translation elongation factor 1 alpha 1, like 1 | gtacttctcaggctgactgtg | acgatcagctgtttcactcc | Normalization |

**Supplementary Information – References**

1 Ma, A. C., Chen, Y., Blackburn, P. R. & Ekker, S. C. TALEN-Mediated Mutagenesis and Genome Editing. *Methods Mol Biol* **1451**, 17-30, doi:10.1007/978-1-4939-3771-4_2 (2016).

2 Neff, K. L. *et al.* Mojo Hand, a TALEN design tool for genome editing applications. *BMC Bioinformatics* **14**, 1, doi:10.1186/1471-2105-14-1 (2013).

3 Cermak, T. *et al.* Efficient design and assembly of custom TALEN and other TAL effector-based constructs for DNA targeting. *Nucleic Acids Res* **39**, e82, doi:10.1093/nar/gkr218 (2011).

4 Bedell, V. M. *et al.* In vivo genome editing using a high-efficiency TALEN system. *Nature* **491**, 114-118, doi:10.1038/nature11537 (2012).

5 Ma, A. C., Lee, H. B., Clark, K. J. & Ekker, S. C. High efficiency In Vivo genome engineering with a simplified 15-RVD GoldyTALEN design. *PLoS One* **8**, e65259, doi:10.1371/journal.pone.0065259 (2013).

6 Meeker, N. D., Hutchinson, S. A., Ho, L. & Trede, N. S. Method for isolation of PCR-ready genomic DNA from zebrafish tissues. *Biotechniques* **43**, 610, 612, 614, doi:10.2144/000112619 (2007).

7 Timonina, K., Kotova, A. & Zoidl, G. Role of an Aromatic-Aromatic Interaction in the Assembly and Trafficking of the Zebrafish Panx1a Membrane Channel. *Biomolecules* **10**, doi:10.3390/biom10020272 (2020).

8 Malicki, J. *et al.* Mutations affecting development of the zebrafish retina. *Development* **123**, 263-273 (1996).

9 Schindelin, J. *et al.* Fiji: an open-source platform for biological-image analysis. *Nat Methods* **9**, 676-682, doi:10.1038/nmeth.2019 (2012).

10 Dougherty, R. in *11th AIAA/CEAS Aeroacoustics Conference (26th AIAA Aeroacoustics Conference)* (2005).

11 Meng, S. *et al.* Targeting retinal dopaminergic neurons in tyrosine hydroxylase-driven green fluorescent protein transgenic zebrafish. *Mol Vis* **14**, 2475-2483 (2008).

12 Gao, Y., Li, P. & Li, L. Transgenic zebrafish that express tyrosine hydroxylase promoter in inner retinal cells. *Dev Dyn* **233**, 921-929, doi:10.1002/dvdy.20416 (2005).

13 Debertin, G. *et al.* Tyrosine hydroxylase positive perisomatic rings are formed around various amacrine cell types in the mammalian retina. *J Neurochem* **134**, 416-428, doi:10.1111/jnc.13144 (2015).

14 Emran, F., Rihel, J. & Dowling, J. E. A behavioral assay to measure responsiveness of zebrafish to changes in light intensities. *J Vis Exp*, doi:10.3791/923 (2008).

15 Pfaffl, M. W., Horgan, G. W. & Dempfle, L. Relative expression software tool (REST) for group-wise comparison and statistical analysis of relative expression results in real-time PCR. *Nucleic Acids Res* **30**, e36, doi:10.1093/nar/30.9.e36 (2002).
